# Retrospektive Analyse von Kindern mit Impfgranulomen

**DOI:** 10.1007/s00105-024-05457-x

**Published:** 2025-01-23

**Authors:** Laura Trefzer, Katrin Kerl-French, Andreas Benedikt Weins, Christina Schnopp

**Affiliations:** 1 Department of Dermatology and Venerology, Medical Center – University of Freiburg, Faculty of Medicine, Hauptstr. 7, 79104 Freiburg, Deutschland; 2 Department of Dermatology and Allergy, Ludwig-Maximilians-Universität, München, Deutschland; 3 Department of Pediatrics and Adolescent Medicine, University of Augsburg, Augsburg, Deutschland; 4 KIDZ SKIN | Practice of Pediatric Dermatology, Ulm, Deutschland; 5 Department of Dermatology and Allergy, Klinikum rechts der Isar, Technical University, München, Deutschland

**Keywords:** Unerwünschte Arzneimittelwirkungen, Kindergesundheit, Kontaktallergie, Juckreiz, Aluminium, Itching nodule, Contact allergy, Child health care, Aluminum, Adverse events

## Abstract

**Hintergrund:**

Impfgranulome sind eine häufige (0,3–1 %), unerwünschte Wirkung von (akzidentell) subkutan verabreichten Impfungen und spezifischen Immuntherapien, die aluminiumhaltige Konjugate enthalten. Die klinische Symptomatik aus juckenden subkutanen Knoten betrifft überwiegend Säuglinge und Kleinkinder an den lateralen Oberschenkeln.

**Ziel der Arbeit:**

Ziel ist es, Dermatologen zu sensibilisieren, diese häufige und harmlose Impfreaktion zu erkennen, um invasive Diagnostik und Verunsicherung von Eltern und Behandlern zu verhindern.

**Material und Methoden:**

Es erfolgen eine retrospektive Analyse von 13 Kindern, die zwischen 2019 und 2023 in der kinderdermatologischen Sprechstunde vorstellig waren, sowie die Herausstellung von Diagnosekriterien und praktischem Handlungsleitfaden unter Einbeziehung der Literatur.

**Ergebnisse und Diskussion:**

Es wurden 13 Kinder (9 Jungen, 4 Mädchen) mit subkutanen indolenten, juckenden Knoten an den Impflokalisationen (11 an Oberschenkeln, 2 an den Oberarmen), die mit einer zeitlichen Latenz von Wochen bis Monaten auftraten, retrospektiv ausgewertet. Die Impfung der Kinder war nach STIKO(Ständige Impfkommission)-Empfehlung erfolgt. Das dokumentierte Auftreten der ersten Impfgranulome war zwischen dem 12. und 36. Lebensmonat (LM). Die 3. Grundimmunisierung der hexavalenten Totimpfung fällt im STIKO-Impfkalender mit der ersten Gabe des Lebendimpfstoffes MMR(Mumps, Masern, Röteln)(‑Varizellen [V]) zusammen (ab 11. LM). Fälschlicherweise kann dies zu der Annahme führen, dass der Lebendimpfstoff ursächlich für die Granulomentwicklung war. Die Aluminiumkonjugation von Totimpfstoffen scheint ein zentraler Auslöser der Granulome zu sein, weitere Suszeptibilitätsfaktoren sind nur unvollständig bekannt. Der Nachweis einer Sensibilisierung auf Aluminium im Epikutantest bringt keinen Zusatznutzen und sollte daher nicht standardmäßig durchgeführt werden. Nach Wochen bis Jahren kommt es zur spontanen Regredienz der Granulome.

**Graphic abstract:**

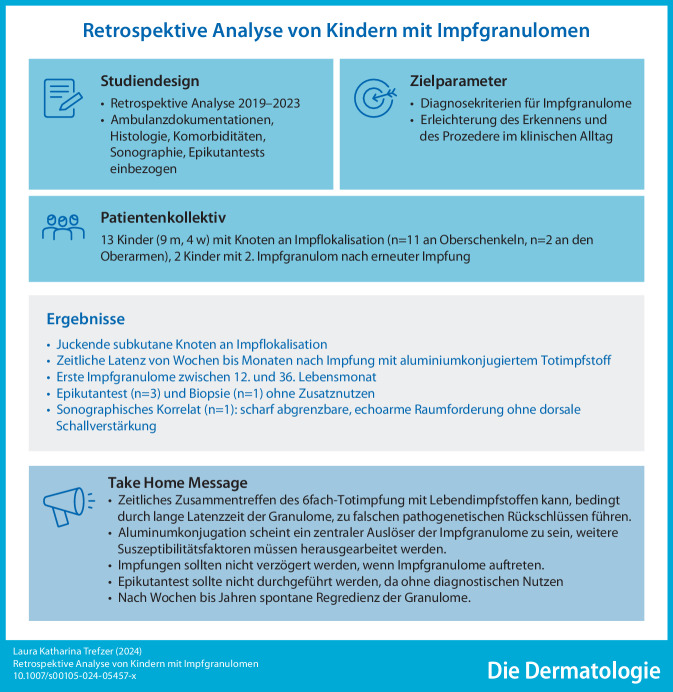

## Abstract (en)

**Background:**

Vaccine granulomas are a common (0.3–1%) adverse event (AE) of (accidentally) subcutaneously administered vaccines and specific immunotherapies containing aluminum conjugates. The clinical symptoms with persistent itching subcutaneous nodules, predominantly affect infants and young children on the lateral thigh.

**Aim:**

To sensitize dermatologists to recognize this common and harmless vaccination AE, in order to prevent invasive diagnostics and confusion among parents and physicians.

**Materials and methods:**

Retrospective analysis of 13 children consulting pediatric dermatology between 2019 and 2023. Identification of diagnostic criteria and guidelines for action, based on the literature.

**Results and conclusion:**

In all, 13 children (9 boys, 4 girls) with subcutaneous indolent but itching nodules at the vaccination sites (11 on the thighs, 2 on the upper arms) with a latency of weeks to months were retrospectively evaluated. The children were vaccinated according to German STIKO (“Ständige Impfkommission”) recommendations. Only inactivated vaccines contain aluminum. The documented occurrence of the first vaccination granulomas was between the 12th and 36th month of life. Regarding the STIKO vaccination calendar, the third immunization with the hexavalent inactivated vaccine coincides with the first administration of the live measles, mumps and rubella (MMR) vaccine (varicella (V)). This may incorrectly lead to the assumption that the live vaccine was the cause of granuloma development. Aluminum conjugation appears to be a central trigger of the granulomas; further susceptibility factors are largely unknown. Diagnosis of sensitization to aluminum through epicutaneous testing has no practical impact and is, therefore, not routinely recommended. After weeks to years, granulomas spontaneously regress.

## Hintergrund und Fragestellung

Impfgranulome sind eine relativ häufige (0,3–1 %), jedoch vielfach nicht erkannte, unerwünschte Wirkung von Impfungen und spezifischen Immuntherapien, die aluminiumhaltige Konjugate enthalten [1, 4, 12]. Die klinische Symptomatik betrifft überwiegend Säuglinge und Kleinkinder und ist charakterisiert durch stark juckende subkutane Knoten an den Impflokalisationen.

Im klinischen Alltag scheint eine Sensibilität für diese Symptomatik nach Impfung zu fehlen und führt so immer wieder zu Verunsicherung bei Behandlern, Patienten und Eltern sowie zu vermeidbarer invasiver Diagnostik und Therapie [3].

## Studiendesign und Untersuchungsmethoden

### Patientenkollektiv

Für diese Studie wurden Patienten, die innerhalb der Jahre 2019 bis 2023 in der kinderdermatologischen Sprechstunde der Klinik für Dermatologie und Allergologie der Technischen Universität München und in der Praxis von Frau Privatdozentin Schnopp in München mit Impfgranulomen vorstellig waren, eingeschlossen. Insgesamt wurde bei 13 Kindern ein Impfgranulom diagnostiziert. Die Datenerhebung erfolgte retrospektiv. Neben Alter und Geschlecht, Lokalisation des Impfgranuloms und subjektiven Symptomen wurden relevante Zusatzinformationen aus der Patientenanamnese, die Histologie, Komorbiditäten, Sonographie und Epikutantest miterfasst.

Die Datenauswertung erfolgte anonymisiert durch Frau PD Schnopp und Dr. Weins unter Berücksichtigung der Deklaration von Helsinki (1975).

### Statistische Aufarbeitung

Die deskriptive statistische Auswertung erfolgte mithilfe von Microsoft Excel®.

## Ergebnisse

Wir berichten von 13 Kindern (9 Jungen, 4 Mädchen), die sich in unserer kinderdermatologischen Sprechstunde mit subkutanen Knoten an den Oberschenkeln bei 11 und an den Oberarmen bei 2 Patienten vorstellten (Tab. 1). Das dokumentierte Auftreten der ersten Impfgranulome war zwischen dem 12. und 36. Lebensmonat (Median 24 Monate, Range 12 bis 36 Lebensmonate, Mittelwert 24 Monate). Bei 2 Kindern wurde ein zweites Impfgranulom an anderer Lokalisation 4 respektive 40 Monate nach dem ersten Granulom dokumentiert (Patient 5, Patient 10).

**Tab. 1:** Patientencharakteristika

P	Geschlecht	Alter bei Auftreten des Granuloms	Impfstoff	Lokalisation	Morphe	Epikutantest mit Aluminiumhexahydrat-Vaseline 2 %	Atopie
1	w	12 Monate	5fach	Oberschenkel rechts lateral	Knoten subkutan	n. d.	–
2	m	36 Monate	MMR	Oberschenkel rechts ventrolateral	Subkutane Knötchen Ekzem	n. d.	Strophulus-artiges Ekzem im Verlauf
3	m	26 Monate	MMRV	Oberschenkel links lateral	Subkutane Knötchen Ekzem Hypertrichose	(+)	–
4	m	24 Monate	MMR	Oberschenkel rechts ventrolateral	Subkutane Knötchen	n. d.	Nummuläres atopisches Ekzem
5	m	20 Monate	5fach + Pneumokokken MMR Hepatitis A	Oberschenkel links lateral	Multiple kleine Knötchen	+++	Prurigoformes Ekzem im Verlauf
		24 Monate	–	Oberschenkel rechts Oberarm links	Abgeheilt Kleine subkutane Knötchen		
6	m	24	MMR	Oberarm rechts lateral	Subkutane Knötchen Ekzem	n. d.	–
7	m	30 Monate	MMRV	Oberschenkel beidseits ventrolateral	Subkutane Knötchen Hyperpigmentierung	n. d.	–
8	w	34 Monate	6fach	Oberschenkel links lateral	Tief subkutanes Knötchen	++	–
9	m	17	MMR	Oberschenkel links lateral	Knoten in der Tiefe Hyperpigmentierung Exkoriationen	n. d.	–
10	w	64 Monate	Meningokokken B	Oberarme beidseits	Derbe Knoten	n. d.	–
		24 Monate	6fach	Oberschenkel lateral beidseits	Plaques in der Tiefe, Ekzem und Hypertrichose		
11	m	24 Monate	MMR	Oberschenkel links ventrolateral	Subkutane Knötchen Ekzem Hyperpigmentierung	n. d.	–
12	m	23 Monate	?	Oberschenkel rechts ventrolateral	Subkutane Knötchen Ekzem	n. d.	–
13	w	18 Monate	?	Oberschenkel rechts ventrolateral	Subkutane Knötchen Ekzem	n. d.	–

*P* Patient, *n.* *d.* nicht durchgeführt, *MMR* Mumps, Masern, Röteln, *MMRV* Mumps, Masern, Röteln, Varizellen, 5fach entspricht Diphtherie, Tetanus, Pertussis, Poliomyelitis, Haemophilus influenzae B, 6fach entspricht Diphtherie, Tetanus, Pertussis, Hepatitis B, Poliomyelitis, Haemophilus influenzae B

Die Diagnosestellung erfolgte klinisch, ein Kind erhielt ergänzend eine sonographische Untersuchung, bei einem Kind wurde schon vor der Erstvorstellung bei uns eine Biopsie aus dem Granulom mit histopathologischer Aufarbeitung durchgeführt, 3 Kinder erhielten einen Epikutantest mit 2 % Aluminiumhexahydrat in Vaseline.

**Abb. 1:**
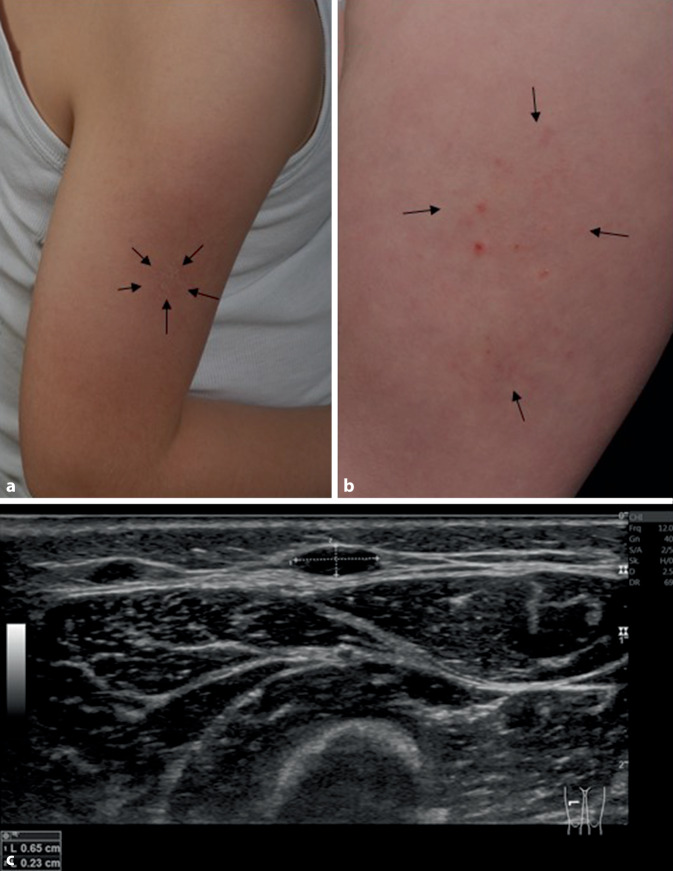
**a** Persistierende Ekzemreaktion (*Pfeile*) der Haut bei einem 7‑jährigen Jungen (Patient 6) nach quadrivalenter Masern-Mumps-Röteln-Impfung in den rechten Oberarm. **b** Persistierende Ekzemreaktion (*Pfeile*) bei einem 6‑jährigen Jungen nach hexavalenter (Diphtherie, Tetanus, Pertussis, Polio, Haemophilus influenzae B, Hepatitis B) Impfung in den linken Oberschenkel. (Patient 8) **c** Sonographie eines Impfgranuloms des ventrolateralen Oberschenkels rechts bei einem 2‑jährigen Jungen (Patient 4)

Die klinischen Charakteristika waren bei allen Kindern tastbare subkutane Knoten, bei 8 Kindern einhergehend mit Ekzemen der darüber liegenden Haut, bei 2 Kindern zeigte sich eine Hypertrichose im betroffenen Areal (Abb. 1a, b) Anamnestisch bestand ein intermittierend auftretender moderater, insbesondere durch Infekte exazerbierter Juckreiz. Die Sonographie des Oberschenkels eines 24 Monate alten Kleinkindes (Patient 4) zeigte als sonographisches Korrelat des tastbaren Nodus eine 12 × 7 × 2 mm messende ovaläre, vom umgebenden subkutanen Gewebe relativ scharf abgrenzbare, echoarme Raumforderung in Abgrenzung zu einer Zyste ohne dorsale Schallverstärkung (Abb. 1c). Der in der Literatur als typisch beschriebene echoarme Randsaum war aufgrund der technischen Bildauflösung und der kleinen Größe des Impfgranuloms nicht eindeutig auszumachen [17].

Alle berichteten Kinder waren entsprechend den STIKO(Ständige Impfkommission)-Empfehlungen geimpft worden. In 7 Fällen trat das Impfgranulom in zeitlichem Zusammenhang mit einer Masern-Mumps-Röteln(MMR)-Impfung auf, in einem Fall mit dem standardisiert eingesetzten 5fach-Totimpfsoff (beinhaltet Diphtherie, Tetanus, Pertussis, Poliomyelitis, Haemophilus influenzae B). Patient 5 erhielt zusätzlich zu der 5fach-Totimpfung noch MMR und einen Pneumokokkenimpfstoff. Patient 8 erhielt den „hexavalenten Totimpfstoff“ (beinhaltet zusätzlich zum 5fach-Impfstoff noch Hepatitis B) und Patient 10 den hexavalenten Impfstoff zeitgleich mit der Pneumokokkenimpfung.

**Abb. 2:**
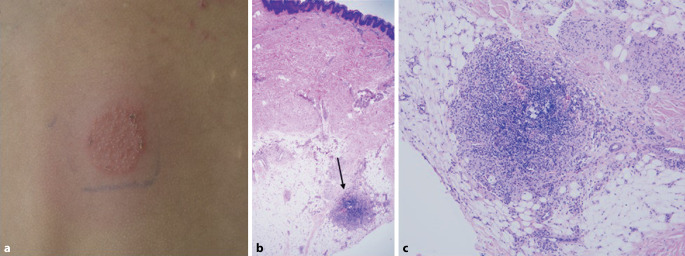
**a** Epikutantest mit Aluminiumhexahydrat-Vaseline 2 % am Rücken bei Patient 5 zeigt Positivität nach 72 h. **b** Histologie von Patient 2: Die 2,5fache Vergrößerung einer Hämatoxylin-Eosin-Färbung einer Biopsie vom Oberschenkel rechts zeigt einen mikronodulären Entzündungsprozess (*Pfeil*), vorwiegend bestehend aus Makrophagen, zahlreichen eosinophilen Granulozyten im Randbereich sowie neutrophilen Granulozyten und Lymphozyten. Es handelt sich dabei um einen charakteristischen, jedoch nicht spezifischen Befund für ein Impfgranulom. **c** 20fache Vergrößerung des Granuloms aus **b**

Drei der Kinder entwickelten im Verlauf eine atopische Dermatitis (Patient 2, 4, 5). Der Epikutantest auf Aluminium wurde bei 3 Patienten durchgeführt und zeigte sich bei 2 positiv (Abb. 2a).

Der histologische Befund der Biopsie bei Patient 2 zeigt einen mikronodulären Entzündungsprozess vorwiegend bestehend aus Makrophagen, zahlreichen eosinophilen Granulozyten im Randbereich sowie neutrophilen Granulozyten und Lymphozyten. Es handelt sich dabei um einen charakteristischen, jedoch nicht spezifischen Befund für ein Impfgranulom (Abb. 2b, c).

Therapeutisch erhielten die Patienten mit Ekzemen über dem Impfgranulom topische Glukokortikosteroide zur symptomatischen Behandlung des Juckreizes (Prednicarbat 0,25 % Creme oder Mometasonfuroat 0,1 % Creme). Die Dauer der Persistenz der Granulome in unserer Kohorte war retrospektiv nicht eruierbar.

## Diskussion

Das Impfgranulom stellt eine klinische Diagnose dar. Die Trias aus indolenten subkutan gelegenen juckenden Knötchen oder Knoten, Impfung an der entsprechenden Lokalisation zusammen mit einer zeitlichen Latenz von Wochen bis Monaten lässt in der Regel eine klinische Diagnose zu [1]. Fakultativ kann sich auf der über dem Granulom befindlichen Haut ein Ekzem zeigen [16]. In unserer Kohorte wurde die Diagnose bei allen Kindern klinisch gestellt, in einem Fall wurde eine Sonographie durchgeführt, in einem Fall war bereits eine Biopsie erfolgt, die die Diagnose bestätigte.

Eine variable Latenzzeit von der Impfung bis zum Auftreten der Granulome und Juckreiz von Wochen bis hin zu mehreren Monaten und Persistenz der Granulome bis hin zu Jahren können die Herstellung des kausalen Zusammenhangs mit einer Impfung erschweren [1].

Differenzialdiagnostisch ist an eine akute irritative oder infektiöse lokale Impfreaktion zu denken, diese geht auch mit Rötung und Schwellung einher, tritt jedoch in unmittelbar zeitlichem Zusammenhang von Stunden bis Tagen mit der Impfung auf [2]. Bei nummulärem oder atopischem Ekzem sind weitere Ekzemherde an den entsprechenden Prädilektionsstellen zu erwarten. Bei multiplen Granulomen ist an ein Immundefizienzsyndrom, bei männlichen Kindern insbesondere auch an die septische Granulomatose zu denken [9, 18].

Der Epikutantest auf Aluminium wurde bei 3 unserer Patienten durchgeführt und zeigte sich bei zweien positiv. In der Literatur sind bei bis zu 94 % der Kinder mit Impfgranulomen positive Ergebnisse im Epikutantest (ECT) gegenüber Aluminium berichtet worden [14]. Die Persistenz der Granulome scheint bei Patienten ohne nachgewiesene Aluminiumkontaktallergie (Typ-IV-Reaktion) kürzer zu sein. Eine spontane vollständige Regredienz der Granulome ist jedoch bei den meisten Patienten unabhängig von einer Aluminiumallergie zu erwarten. Die in der Literatur beschriebene mediane Dauer der Persistenz beträgt 22 Monate [1, 16]. Die Aluminiumsensibilisierung ist häufig nach Jahren nicht mehr nachweisbar [3, 6, 7, 19], kann jedoch auch bei Kindern ohne Impfgranulome (8 %) auftreten [4]. Die Aluminiumepikutantestung ist folglich weder obligat für die Diagnosestellung noch hat sie prognostisch eindeutige Aussagekraft, kann jedoch zu Verunsicherung der Patienten bezüglich weiterer Impfungen führen [14]. Im klinischen Alltag ist daher aus unserer Sicht von der Epikutantestung gegenüber Aluminium bei klassischen Impfgranulomen abzusehen.

In Deutschland werden die Impfempfehlungen durch die Ständige Impfkommission (STIKO) des Robert Koch-Institutes (RKI) herausgegeben und im Rahmen des Impfkalenders aktualisiert veröffentlicht [20]. Ab dem Alter von 6 Wochen ist der erste empfohlene injizierbare Impfstoff der hexavalente Diphtherie‑, Tetanus‑, Pertussis‑, Hepatitis-B-, Poliomyelitis‑, Haemophilus-influenzae-B-Impfstoff, der zur intramuskulären Injektion bevorzugt in den M. vastus lateralis vorgesehen ist [20]. Es sind Impfseren von 3 verschiedenen Herstellern auf dem europäischen Markt erhältlich, diese enthalten laut Fachinformationen der Hersteller pro Impfdosis als Konjugator 0,5–0,6 mg Aluminium (Al3+). Aluminium ist in vielen verschiedenen Totimpfstoffen (z. B. auch Meningokokken B, Frühsommermeningoenzephalitis) enthalten und auch Bestandteil der subkutanen spezifischen Immuntherapie, nicht jedoch in Lebendimpfstoffen [8]. Wird entsprechend dem STIKO-Impfkalender geimpft, so fällt die 3. Grundimmunisierung der hexavalenten Totimpfung zeitlich mit der ersten Gabe des Lebendimpfstoffes MMR(‑Varizellen [V]) zusammen. Dies kann fälschlich zu der Annahme führen, dass der Lebendimpfstoff ursächlich für die Granulomentwicklung war.

Eine Sensibilisierung auf Aluminium ist weder notwendig noch hinreichend für die Entwicklung von Impfgranulomen. Daher scheinen für die Granulomentwicklung weitere Suszeptibilitätsfaktoren eine Rolle zu spielen [15]. Die bis dato bekannten prädisponierenden Faktoren wie weibliches Geschlecht, familiäre Prädisposition für Granulome und Frühgeburtlichkeit sind keine vermeidbaren Faktoren [12]. Aktuelle Untersuchungen zeigen, dass das Vorliegen einer atopischen Dermatitis mit einer höheren Wahrscheinlichkeit für Impfgranulome einhergeht [13]. Auch in unserer Kohorte entwickelten 3 der Patienten im Verlauf eine atopische Dermatitis.

Histologisch lässt sich bei Exzision der Granulome ein gemischtzelliges Infiltrat mit Lymphozyten, Histiozyten und Eosinophilen und typischen amphophilen Granula in den beteiligten Histiozyten nachweisen, die als charakteristisch für Aluminiumgranulome angesehen werden [10]. Pathomechanistisch wird daher die Impfgranulomentwicklung am ehesten auf die Konjugation mit Aluminium im Sinne eines Fremdkörpergranuloms zurückgeführt. Eine Impfgranulomentwicklung nach Lebendimpfungen ohne Aluminiumkonjugator wurde auch in der Literatur berichtet, ist aber zumindest deutlich seltener zu beobachten als bei konjugierten Impfstoffen [4, 14]. Nach aktuellem wissenschaftlichem Stand ist davon auszugehen, dass Granulomentwicklung ein Mechanismus ist, den Körper vor Fremdmaterial zu schützen. Interleukin-10 und Tumornekrosefaktor-alpha werden dabei als zentrale Zytokine für die Granulomentwicklung und deren Persistenz angesehen [5]. Der Grund dafür, dass der Großteil der geimpften Kinder keine Granulome entwickelt, ist nicht geklärt, ebenso wenig, wieso ein Drittel der Kinder die Granulome bereits nach der ersten Impfung entwickelt [3, 14] und warum bei wiederholter Gabe eines aluminiumhaltigen Impfstoffs nur in Einzelfällen neue Granulome entstehen [3]. Eine höhere Wahrscheinlichkeit bei subkutaner im Gegensatz zu intramuskulärer Injektion ist naheliegend, die Datenlage dazu aber schwach [4].

In der Regel sind Impfgranulome im Verlauf spontan regredient [16]. Bis zur vollständigen Abheilung eines Impfgranuloms kann es mehrere Jahre dauern, daher ist eine eingehende Aufklärung der Eltern bezüglich des zeitlichen Verlaufs wichtig. Von operativer Entfernung, einhergehend mit operativen Risiken sowie konsekutiver Narbenbildung, ist aufgrund des selbstlimitierenden Verlaufs abzusehen. Differenzialdiagnostisch können Abszesse, Fremdkörpergranulome und maligne Prozesse sonographisch abgegrenzt werden. Eine symptomatische Therapie des Juckreizes kann mit topischen Glukokortikoiden oder bei Erwachsenen mit topischem Capsaicin erfolgen [11].

## Limitationen

Unsere Studie weist Limitationen auf. So ist das Patientenkollektiv mit 13 Kindern vergleichsweise klein, die Geschlechterverteilung entspricht nicht der Mädchendominanz in der Literatur. Zudem ergibt sich aus dem Studiendesign der retrospektiven Analyse die Möglichkeit eines Recallbias, sodass die Patienten sich ggf. nicht mehr vollständig an den Beschwerdebeginn erinnerten. Aufgrund der langen Latenz zwischen Impfung und Granulomentstehung wurde in 7 Fällen ein zeitlicher Zusammenhang mit der MMR-Impfung berichtet und die zeitgleich oder in engem zeitlichem Zusammenhang verabreichte 3. Impfung mit dem 6fach-Impfstoff nicht berichtet, ebenso wenig konnten wir aus den Akten exakt ermitteln, welche Impfstoffe (Hersteller) genau appliziert wurden.

## Fazit für die Praxis

- Impfgranulome sind anhand der typischen Trias aus indolenten subkutan gelegenen juckenden Knötchen oder Knoten sowie dokumentierter Impfung in den letzten Monaten an der entsprechenden Lokalisation in Zusammenschau mit der zeitlichen Latenz von Wochen bis Monaten klinisch zu diagnostizieren.
- Die Sonographie ist ein wichtiges Instrument zur differenzialdiagnostischen Abgrenzung.
- Eine Fremdkörperreaktion oder Typ-IV-Allergie gegenüber Aluminium scheint der Mechanismus für die Entwicklung von Impfgranulomen zu sein.
- Eine Epikutantestung gegenüber Aluminium sollte aufgrund der fehlenden klinischen Relevanz nicht durchgeführt werden.
- Das Impfgranulom ist eine harmlose, wenn auch durch den einhergehenden Juckreiz störende, unerwünschte Wirkung von aluminiumhaltigen Impfstoffen.
- Da der Nutzen der Impfungen überwiegt und die Impfgranulome für gewöhnlich komplett regredient sind, sollte nicht auf indizierte Impfungen verzichtet werden.

## Data Availability

Die Daten, die die Ergebnisse dieser Studie untermauern, sind auf Anfrage bei der korrespondierenden Autorin erhältlich.
